# Supplementary material for: Wearable High Voltage Compliant Current Stimulator for Restoring Sensory Feedback
Source: Micromachines (Basel). 2023 Mar 30;14(4):782. doi: 10.3390/mi14040782 (PMC10143515; doi:10.3390/mi14040782)
Supplement: Supplementary file 1 [file micromachines-14-00782-s001.zip › micromachines-2248551-supplementary.pdf]

Wearable High Voltage Compliant Current Stimulator For Sensory Feedback Restoring

Riccardo Collu[1], Roberto Paolini[2], Martina Bilotta[2], Andrea Demofonti[2], Francesca Cordella[2], Loredana Zollo[2] and Massimo Barbaro[1]*

[1] Department of Electrical and Electronics Engineering, University of Cagliari, Piazza D'Armi, 09123 Cagliari, Italy; riccardo.collu@unica.it; massimo.barbaro@unica.it

[2] Research Unit of Advanced Robotics and Human-Centred Technologies (CREO Lab), Università Campus Bio-Medico di Roma, Rome, Italy; r.paolini@unicampus.it; martina.bilotta@alcampus.it; a.demofonti@unicampus.it; f.cordella@unicampus.it; l.zollo@unicampus.it

* Correspondence massimo.barbaro@unica.it

Results and Discussion

Measurements reported in results and discussion were reproduced on 1.05kΩ and 4.7kΩ resistive load as reported in

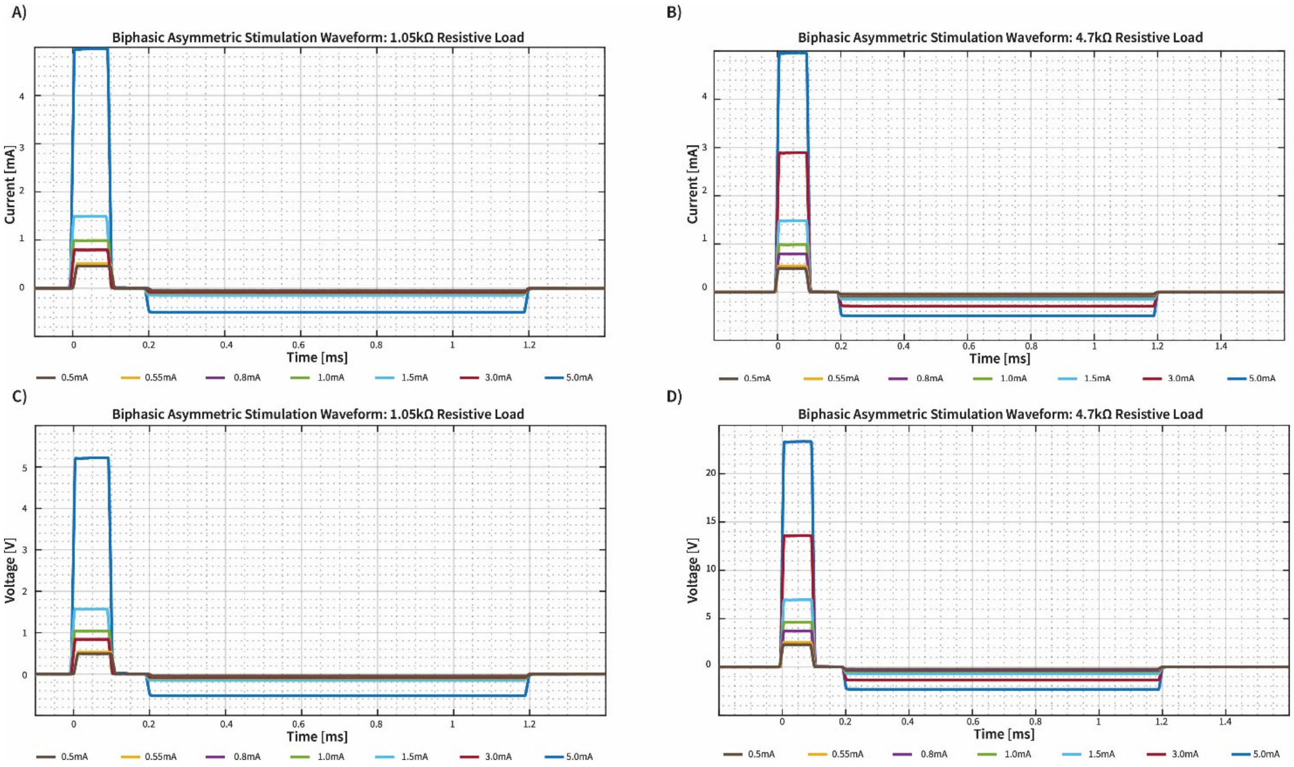

Figure S1 Waveform generation capability using Biphasic asymmetric waveforms with different amplitude and pulse width 100µs were tested on 1.05kΩ resistive load (A-C) and on 4.7kΩ (B-D)

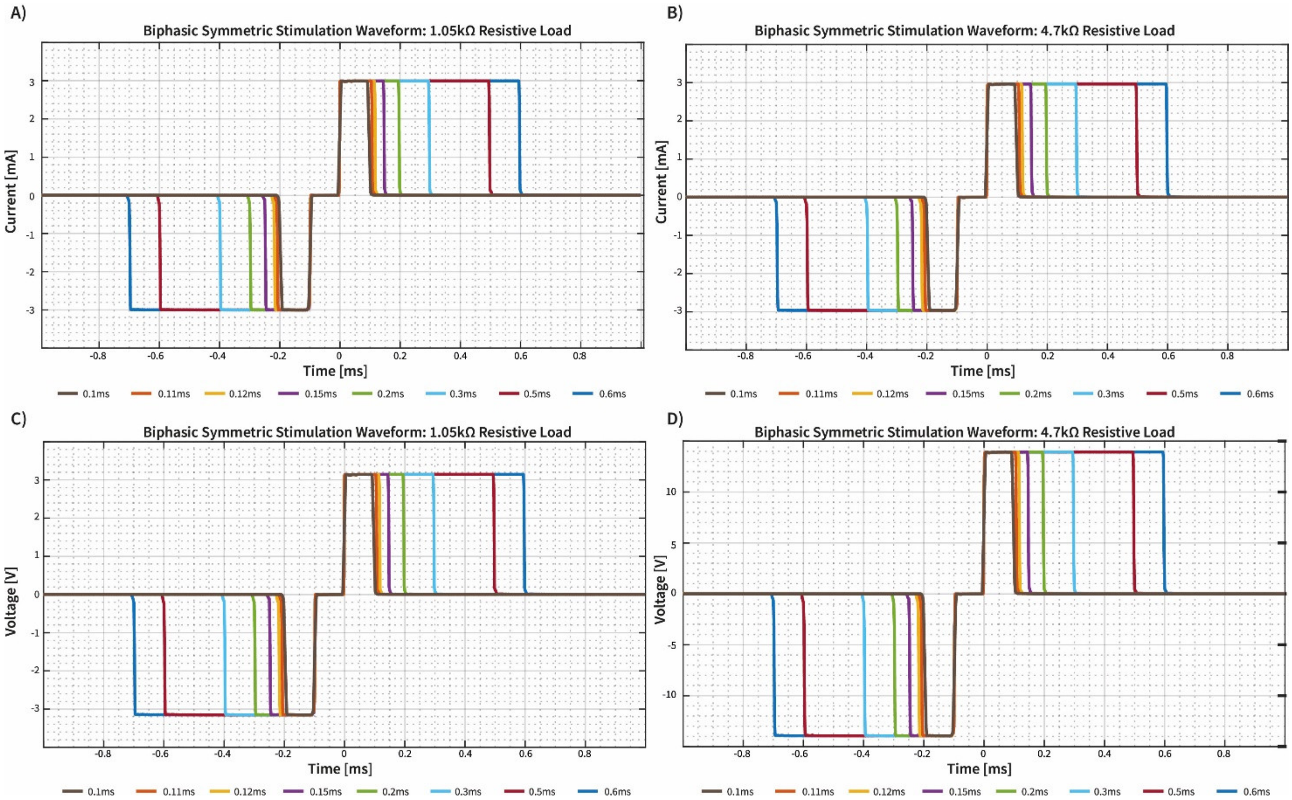

Figure S2 Waveform generation capability using Biphasic symmetric waveforms with different pulse width and amplitude 3mA were tested on 1.05kΩ resistive load (A-C) and on 4.7kΩ (B-D)
